# Differential molecular programs of cutaneous anaplastic large cell lymphoma and CD30-positive transformed mycosis fungoides

**DOI:** 10.3389/fimmu.2023.1270365

**Published:** 2023-09-18

**Authors:** Pan Lai, Fengjie Liu, Xiangjun Liu, Jingru Sun, Yang Wang

**Affiliations:** ^1^ Department of Dermatology and Venereology, Peking University First Hospital, Beijing, China; ^2^ Beijing Key Laboratory of Molecular Diagnosis on Dermatoses, Beijing, China; ^3^ National Clinical Research Center for Skin and Immune Diseases, Beijing, China; ^4^ Department of Dermatology, Sun Yat-sen Memorial Hospital, Sun Yat-sen University, Guangzhou, China; ^5^ Department of Dermatology, Shandong University Qilu Hospital, Jinan, China

**Keywords:** cutaneous anaplastic large cell lymphoma, CD30-positive transformed mycosis fungoides, gene expression profile, immunohistochemistry algorithm, differential diagnosis

## Abstract

**Background:**

Discriminating between cutaneous anaplastic large cell lymphoma (cALCL) and CD30-positive transformed mycosis fungoides (CD30+ TMF) is challenging, particularly when they arise in the context of pre-existing mycosis fungoides. The development of molecular diagnostic tools was hampered by the rarity of both diseases and the limited understanding of their pathogenesis.

**Methods:**

In this study, we established a cohort comprising 25 cALCL cases and 25 CD30+ TMF cases, with transcriptomic data obtained from 31 samples. We compared the clinicopathological information and investigated the gene expression profiling between these two entities. Furthermore, we developed an immunohistochemistry (IHC) algorithm to differentiate these two entities clinically.

**Results:**

Our investigation revealed distinct clinicopathological features and unique gene expression programs associated with cALCL and CD30+ TMF. cALCL and CD30+ TMF displayed marked differences in gene expression patterns. Notably, CD30+ TMF demonstrated enrichment of T cell receptor signaling pathways and an exhausted T cell phenotype, accompanied by infiltration of B cells, dendritic cells, and neurons. In contrast, cALCL cells expressed high levels of HLA class II genes, polarized towards a Th17 phenotype, and exhibited neutrophil infiltration. An IHC algorithm with BATF3 and TCF7 staining emerged as potential diagnostic markers for identifying these two entities.

**Conclusions:**

Our findings provide valuable insights into the differential molecular signatures associated with cALCL and CD30+ TMF, which contribute to their distinct clinicopathological behaviors. An appropriate IHC algorithm could be used as a potential diagnostic tool.

## 1 Introduction

Mycosis fungoides (MF) is the most common type of cutaneous T cell lymphoma (CTCL) and typically follows an indolent clinical course (1). However, 20% to 55% of MF patients undergo large cell transformation (LCT), marked by aggressive skin tumor progression and resistance to standard treatments, resulting in a dismal 5-year survival rate of less than 20% (2–5). Histologically, LCT is defined by the presence of more than 25% of MF cells being four times larger than a normal lymphocyte or the presence of clusters of large cells (3). 15.5% to 39% of transformed MF cells express CD30 antigen (4, 6). This identical morphology makes it difficult to differentiate CD30-positive transformed MF (CD30+ TMF) from cutaneous anaplastic large cell lymphoma (cALCL), which belongs to the CD30+ lymphoproliferative disorders (CD30+ LPDs) subgroup of CTCL and has a more favorable 5-year survival rate exceeding 80% (1, 7). cALCL can coexist with MF lesions, which further complicates the differentiation from CD30+ TMF (8). Accurate diagnosis is essential because the prognosis and treatment of these two diseases are distinct.

Differential diagnosis is often based on clinical presentation, but clinical indicators are often confounded when cALCL and TMF coexist with skin lesions of MF, thus limiting their discriminative value (9, 10). Due to the rarity of cALCL and CD30+ TMF, the pathogenetic mechanisms of both diseases remain largely unknown. The unique molecular characteristics have been investigated primarily through single aberrant molecules for a long time. IRF4 translocation detected by fluorescence *in situ* hybridization was proposed to be highly specific for cALCL but was also found in some cases of TMF (11–13). SATB1/CD30 colocalization detected by immunofluorescence staining was not readily applicable in routine clinical practice (14). Immunostaining studies showed a notable disparity in GATA3 expression between the two diseases but lacked robust sensitivity and specificity (15, 16).

To comprehensively understand the biological distinction between cALCL and CD30+ TMF, a systematic comparison of their whole molecular landscape is needed. Unsupervised clustering of array comparative genomic hybridization data suggested different genetic bases for the two diseases (17). However, cALCL has been frequently studied alongside other peripheral anaplastic large cell lymphomas (18, 19), and CD30+TMF has been combined with advanced MF in previous studies (20). A direct comparison of the molecular profiles between these two entities is currently lacking.

In our study, we assembled a cohort comprising 25 cALCL cases and 25 CD30+ TMF cases with long-term follow-up. We analyzed the clinicopathologic characteristics and gene expression profiles of cALCL and CD30+ TMF. Additionally, we developed an immunohistochemistry (IHC) algorithm to differentiate between these two entities.

## 2 Materials and methods

### 2.1 Patient collection

The study was based on the TreAtments and outComes in paTients with prImary CutAneous Lymphoma (TACTICAL) database which was established in August 2009 as the registry of patients with cutaneous lymphoma identified in the Skin Lymphoma Clinic in Peking University First Hospital (a tertiary referral center for skin lymphoma in China) (21). Patients with a definite diagnosis of cutaneous lymphoma according to the 2005 World Health Organization- European Organization for Research and Treatment of Cancer (WHO-EORTC) consensus classification (22) and/or its 2018 updated version (1) were enrolled in our database. In MF cases, tumor cell expression of CD30 and large cell transformation were determined based on pathology reports recorded in the database. We screened patients with detailed clinical and histopathological information. Patients did not receive treatment before undergoing biopsies. Moreover, their tumor tissue specimens contained at least 70% neoplastic T-cell infiltration. Sufficient formalin-fixed paraffin-embedded (FFPE) skin tumor biopsies were required for immunohistochemical experiments or published bulk RNA sequencing data were available for re-analysis. Finally, we included 25 cALCL cases and 25 CD30+ TMF cases, containing 52 samples. The mean duration of follow-up was 39.4 months [median = 30.5 (1-143)] in the cALCL group and 22.4 months [median = 17 (3-92)] in the CD30+TMF group. Overall survival (OS) was calculated from the date of initial diagnosis until the date of all-cause mortality. Progression-free survival (PFS) was calculated from the date of initial diagnosis until the first date when the criteria for disease progression were met or until death from any cause (23). Disease progression was defined as progression to a more advanced TNMB classification (excluding a change from Tla or T2a to T1b or T2b, respectively) or death owing to the disease (24). Detailed patient information is provided in **Supplementary Table 1**. Comparison of clinical and histopathological data was carried out by the descrTable function in compareGroup R package.

### 2.2 Data collection and preprocessing

We utilized previously published transcriptome data from our group (GSE168508, HRA000166, and GSE109620) and performed a re-analysis of 16 CD30+ TMFs and 15 cALCLs. The raw paired-end reads underwent trimming and quality control using fastp (25). Subsequently, the clean reads were aligned to the human reference genome hg38 in orientation mode using HISAT2 software (26). The mapped reads for each sample were assembled using StringTie (27) in a reference-based approach. To eliminate non-biological variations, we employed the RUVSeq package in R software (28). Upper-quartile normalization was performed and then the deviance residuals were computed from the generalized linear model (GLM) fit. All the genes were used to estimate the factors of unwanted variation. The debatched results were further assessed using principal component analysis (PCA). Additionally, we obtained ArrayExpression data (GSE14879) from the Gene Expression Omnibus (GEO) database (18), which included 7 isolated cALCL cells and 8 normal CD4-positive T cells, for further analysis and comparison.

### 2.3 Differential expression analysis and gene set enrichment

Differential expression analysis was performed between the cALCL and CD30+TMF groups by DESeq2 (29). Differential gene expression analysis between cALCL tumor cells and normal T cells was conducted using the Limma R package (30). P-values were adjusted using the Benjamin-Hochberg method to control for false discovery rates. Gene counts were normalized to transcripts per million (TPM). To visualize the differential expression patterns, gene expression data were plotted using the ggplot2 R package, and heatmaps were generated using the ComplexHeatmap or Pheatmap R packages. The scale function in the base R package was used to normalize gene expression before plotting the heatmap. Gene set enrichment analysis (GSEA) was performed by feeding log2 fold changes computed by DESeq2 into the GSEA preranked module of the GenePattern server (http://www.genepattern.org/) (31).

### 2.4 Single-sample gene set enrichment analysis (ssGSEA)

The ssGSEA program, implemented in the R package gsva (32), was used to calculate the enrichment scores for each T cell phenotype term. T cell status markers were referenced from the previously studies (33–36), including Th1 (IFNG, IL12A, IL18, STAT1, STAT4, TBX21), Th2 (IL13, IL7R, STAT6, GATA3), Th17 (IL21, RORC, IL17RA, IL6, STAT3, IL1A, RORA), Tex (PDCD1, TIGIT, LAYN, LAG3, CTLA4, HAVCR2, ENTPD1, CXCL13, TOX, TOX2), cytotoxic T cell (CST7, GZMA, GZMB, IFNG, NKG7, PRF1).

### 2.5 Regulon-activity analysis

Regulator-target associations were inferred using the RTN package of R software, which utilizes mutual information and the algorithm for reconstructing accurate cellular networks (ARACNe) (37, 38). Transcription factors meeting the following criteria were selected for transcriptional network inference: 1) reported as a human transcription factor by Lambert, et al. (39); 2) differentially expressed when compared cALCL with CD30+ TMF with a certain level of higher expression (absolute log2 fold change ≥1, baseMean computed by DESeq2 >200, **Supplementary Table 2**). The association of regulon activity and disease was calculated with the ranked gene list with log2 fold change through a two-tailed GSEA implemented in the RTN package of R. The regulators with absolute regulon activity> 1.6 and p-value< 0.001 were further analyzed for their regulatory effect on each sample. Both the regulon activity and regulator expression levels (log2 (TPM+1)) of individual samples were visualized using the ComplexHeatmap R package.

### 2.6 Cellular infiltration estimation

Cellular infiltration in the tumor microenvironment was estimated using the xCell algorithm (40). Immune cells, excluding T cell types, and stromal cells involved in skin lesions from 64 cell types in the xCell dataset were used in the calculation. The cell-type proportions were rescaled 0 and 1, by subtracting the minimum value and dividing by the difference between the maximum and minimum values.

### 2.7 Immunohistochemistry

Paraffin-embedded sections were deparaffinized and rehydrated. After antigen retrieval and endogenous peroxidase inactivation and blocking, slides were incubated with antibodies against BATF3 (Abcam, ab3022568) at a dilution of 1:500, and TCF7 (Cell Signal Technology, C63D9) at a dilution of 1:200, respectively. Goat anti-rabbit IgG HRP H&L (HRP) secondary antibody (Abcam, Ab205718) at a dilution of 1:5000 was used. Slides were scanned by a Nano-Zoomer microscopic slide scanner (Hamamatsu Photonics, Hamamatsu, Japan). Each staining was evaluated by visual estimation as previously reported (41) and verified by cell count performed in 3 randomized fields of view, and the average percentage of positive tumor cells stained was used for analysis.

### 2.8 Statistical analysis

Statistical analysis was performed using R software (version 4.3.0) in this study. Various statistical tests were employed depending on the specific analysis and research question. The chi-square test, Fisher’s exact test, log-rank test, Wilcoxon test, Spearman rank correlation test, and Pearson correlation test were used as appropriate. Detailed statistic methods can be found in the corresponding Figure Legends.

## 3 Results

### 3.1 Clinical and pathological characteristics of cALCL and CD30+ TMF

**Figure 1:**
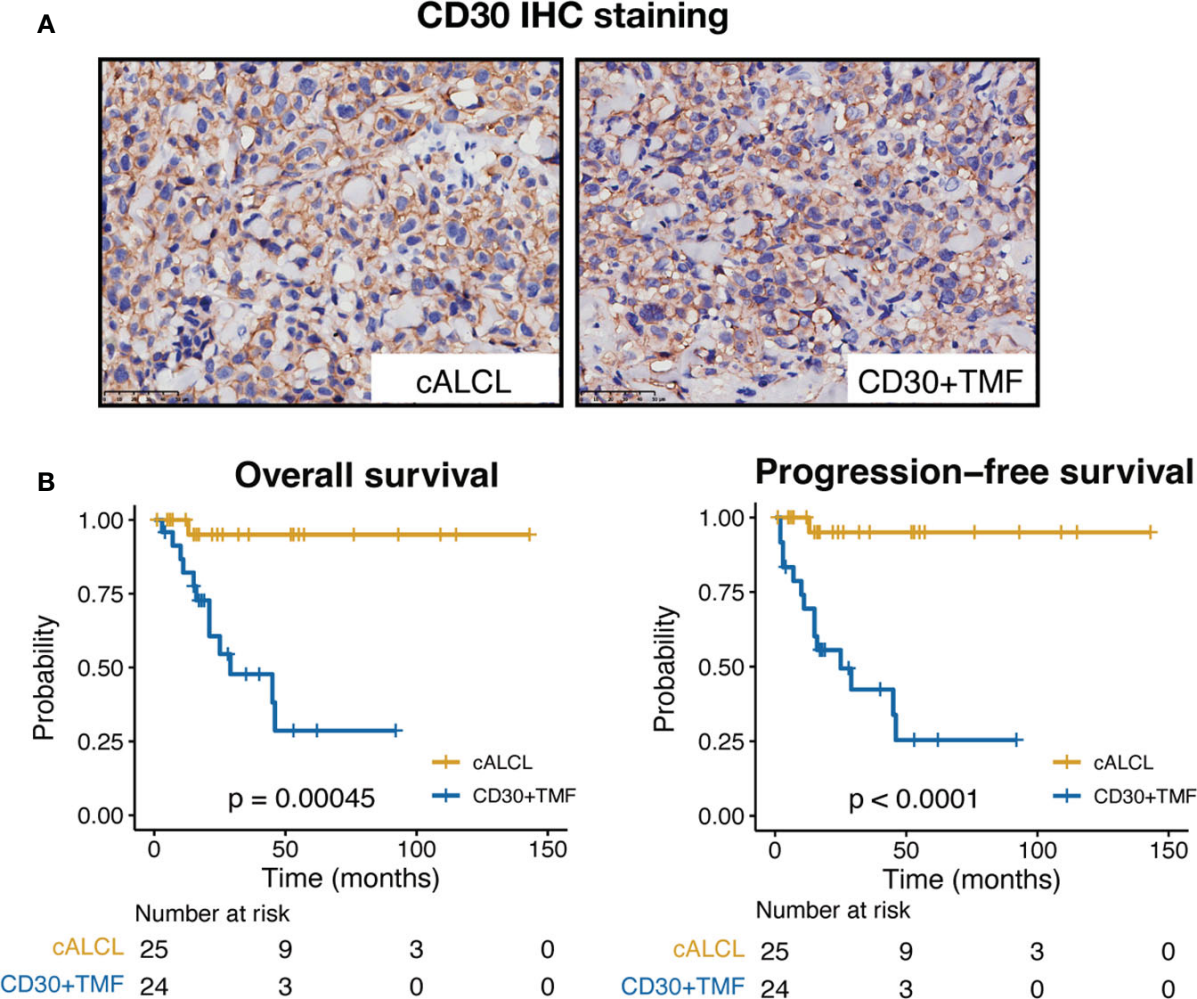
CD30 immunostains and clinical outcomes of cALCL and CD30+ TMF groups. **(A)** Representative images of CD30 IHC staining in biopsy specimens from cALCL and CD30+ TMF patients. IHC, immunohistochemistry. Original magnification ×400. Scale bar = 50 μm. **(B)** Kaplan-Meier survival analysis of overall survival and progression-free survival in 25 cALCL patients and 25 CD30+ TMF patients. Statistical significance was determined by the log-rank test.

A total of 25 cALCL cases and 25 CD30+ TMF cases were included in this study, and the representative CD30 immunostains of the two groups are shown (**Figure 1A**). We compared the clinical and histopathologic data of two groups: cALCL and CD30+ TMF (**Table 1**). cALCL tended to relapse (p < 0.001) but also regressed spontaneously in 32% of cases (p = 0.023), whereas CD30+ TMF showed a more aggressive course with a tendency to progress (p < 0.001). cALCL lesions were solitary or locally disseminated. In contrast, CD30+ TMF lesions were regionally disseminated with involvement of the trunk and lower extremities (both p < 0.001). Notably, pruritus was a common symptom in CD30+ TMF patients (87.0%). In contrast, cALCL patients reported it less frequently (p < 0.001). More CD30+ TMF patients had abnormal serum levels of lactate dehydrogenase (LDH) than cALCL patients (p = 0.001). Histologically, TMF cells were more epidermotropic than anaplastic large cells in cALCL (p = 0.014). TMF lesions showed more prominent CD20-positive B-cell infiltration (p = 0.023), while neutrophils were absent. In contrast, 8 of 22 cALCL specimens demonstrated neutrophil infiltration (p = 0.001). The degree of epidermal hyperplasia, eosinophil infiltration, CD8-positive immunostaining, and percentage of Ki-67-positive cells were comparable in both diseases.

**Table 1:** Comparative clinicopathological data of cALCL group and CD30+ TMF group.

	cALCL (n=25)	CD30+TMF (n=25)	P-value
**Gender**			0.086
Male	18(72.0%)	11(44.0%)	
Female	7(28.0%)	14(56.0%)	
**Trunk involvement**			**<0.001**
Yes	11(45.8%)	24(100%)	
No	13(54.2%)	0(0.00%)	
**Low-limb involvement**			**<0.001**
Yes	10(43.5%)	23(95.8%)	
No	13(56.5%)	1(4.17%)	
**Number and distribution of lesions**			**<0.001**
Solitary	6(24.0%)	0(0.00%)	
Local-disseminated	10(40.0%)	0(0.00%)	
Regional-disseminated	9(36.0%)	25(100%)	
**Ulceration**			**0.032**
Yes	18(72.0%)	9(37.5%)	
No	7(28.0%)	15(62.5%)	
**Relapse**			**<0.001**
Yes	18(72.0%)	4(16.0%)	
No	7(28.0%)	21(84.0%)	
**Disease progression**			**<0.001**
Yes	1(4.00%)	25(100%)	
No	24(96.0%)	0(0.00%)	
**Spontaneous regression**			**0.023**
Yes	8(32.0%)	1(4.00%)	
No	17(68.0%)	24(96.0%)	
**Pruritus**			**<0.001**
Yes	6(24.0%)	20(87.0%)	
No	19(76.0%)	3(13.0%)	
**LDH level**			**0.001**
Elevated	0(0.00%)	11(57.9%)	
Normal	16(100%)	8(42.1%)	
**Epidermotropism**			**0.014**
Yes	9(40.9%)	20(80.0%)	
No	13(59.1%)	5(20.0%)	
**Epidermal hyperplasia**			0.402
Yes	10(45.5%)	7(29.2%)	
No	12(54.5%)	17(70.8%)	
**Eosnophil infiltration**			1
Yes	10(50.0%)	13(54.2%)	
No	10(50.0%)	11(45.8%)	
**Neutrophil infiltration**			**0.001**
Yes	8(36.4%)	0(0.00%)	
No	14(63.6%)	25(100%)	
**CD20 immunostains**			**0.023**
Positive	2(11.1%)	8(50.0%)	
Negative	16(88.9%)	8(50.0%)	
**CD8 immunostains**			0.122
Positive	7(50.0%)	13(81.2%)	
Negative	7(50.0%)	3(18.8%)	
**Ki-67(%)**	80.0[50.0;90.0]	50.0[35.0;80.0]	0.097

Statistical significance was determined by the chi-squared test or Fisher’s exact test. LDH, the serum levels of lactate dehydrogenase. P-values of indicators significantly associated with disease (p-value ≤ 0.05) are bolded.

In terms of prognosis, the cALCL group showed significantly favorable outcomes, with both overall survival (OS) and progression-free survival (PFS) rates estimated to be 95% at the end of follow-up (median follow-up time = 30.5 months, range = 1 to 143 months). In contrast, the CD30+ TMF group showed dismal outcomes during the follow-up period (median follow-up time = 17 months, range = 3 to 92 months), with an OS rate of 28.6% and a PFS rate of 25.4% at the end of follow-up (**Figure 1B**). Therefore, we found notable differences in clinicohistopathologic features and patient prognosis between cALCL and CD30+ TMF.

### 3.2 cALCL and CD30+ TMF exhibit distinct molecular programs

**Figure 2:**
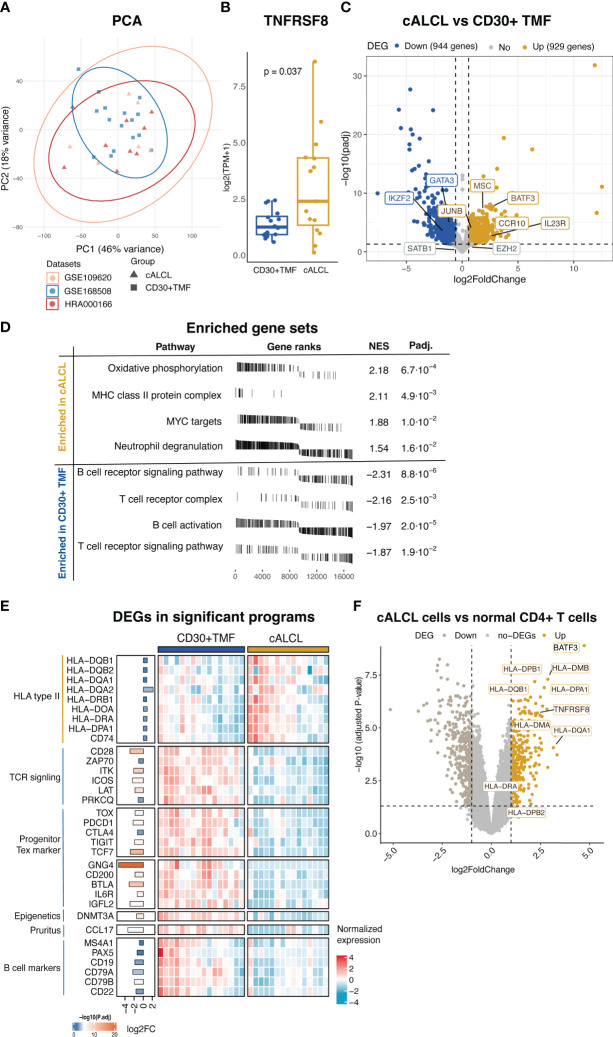
Differential transcriptional programs in cALCL and CD30+TMF groups. **(A)** Principal component analysis on the merged data sets of cALCL and CD30+ TMF transcriptomics after normalization and batch correction. **(B)** Expression of TNFRSF8 in CD30+TMF and cALCL compared by Wilcoxon test. **(C)** Volcano plot of DEGs (fold change ≥ 1.5 or ≤ -1.5 and adjusted P-value < 0.05) by comparing the cALCL group with the CD30+TMF group. **(D)** The plots table of enrichment graphs included enriched gene sets in the cALCL group and CD30+ TMF group. NES, normalized enrichment score, padj, adjusted P-value. **(E)** Heatmap includes differentially expressed genes related to significant programs. Bar lengths and color represent log2 fold change and -log10 (adjusted P value), respectively, on the left side of the heatmap. **(F)** Volcano plot of DEGs (fold change ≥ 2 or ≤ -2 and adjusted P value < 0.05) comparing cALCL cells with normal CD4+ T cells. The differentially expressed HLA class II genes were indicated. PCA, principal component analysis, DEGs, differentially expressed genes, TCR, T cell receptor, Tex, exhausted T cell.

Next, we performed a comparative analysis of the transcriptional atlas between 15 cALCL lesions and 16 CD30+ TMF lesions to gain insights into the molecular events underlying their distinct clinical behaviors. The principal component analysis (PCA) showed no systematic dataset-specific bias in the merged transcriptome (**Figure 2A**). Transcript levels of *TNFRSF8* (the gene encoding CD30) were higher in cALCL compared to CD30+ TMF (**Figure 2B**). In addition, we identified 929 up-regulated genes and 944 down-regulated genes when comparing the cALCL group with the CD30+ TMF group (fold change ≥ 1.5 or ≤ 1.5 and P-value < 0.05), demonstrating characteristic transcriptional profiles in the two entities (**Figure 2C**, **Supplementary Table 2**). Notably, genes previously reported to be associated with each disease were differentially expressed between the two groups. We found that BATF3 and JUNB showed higher expression in cALCL compared to CD30+ TMF (**Figure 2C**), and previous reports have highlighted their essential role in the pathogenesis of cALCL (19, 42, 43). In contrast, both EZH2 and SATB1 showed comparable expression levels in the two groups (**Figure 2C**), reflecting their reported roles in both diseases (14, 44–46). A group of genes (*IL23R*, *CCR10*, *MSC*) were reported to be upregulated in cALCL (47), and we found their higher expression in cALCL compared to CD30+ TMF (**Figure 2C**). We detected GATA3, previously reported as a diagnostic candidate to differentiate cALCL from CD30+ TMF (15), exhibited distinct expression in CD30+ TMF in our data (**Figure 2C**). IKZF2, with its reported role in LCT (48), showed higher expression in CD30+ TMF when compared (**Figure 2C**).

Next, we examined the distinct cytopathogenic programs with the differentially expressed genes between the two entities. GSEA results showed that cALCL lesions were enriched in MHC class II protein complex, oxidative phosphorylation, MYC targets, and neutrophil degranulation, whereas CD30+ TMF lesions were enriched in T cell receptor (TCR) signaling pathway and B cell receptor signaling pathway/B cell activation (**Figure 2D**, **Supplementary Table 3**). Consistently, we found that several HLA type II genes were highly expressed in cALCL compared to CD30+ TMF (**Figure 2E**). The higher expression of HLA type II in cALCL lesions could be the result of infiltrating APCs, reactive T cells, or their expression in malignant T cells (49–51). To gain further insight, we analyzed the microarray data including isolated cALCL malignant cells and normal CD4+ T cells in a published dataset (18) and found that HLA class II genes were highly expressed in cALCL cells compared to normal CD4+ T cells (**Figure 2F**). Oxidative phosphorylation emerged as a significant feature of the cALCL group driven collectively by a set of genes, although no individual genes were found to be highly differentially expressed genes (**Figure 2D**, **Supplementary Table 2**). This suggests that the regulation of this process in cALCL may be more complex, involving the coordinated expression and interaction of multiple genes rather than the upregulation or downregulation of a few specific genes. Compared to cALCL, CD30+ TMF showed a higher expression of several genes involved in the TCR signaling pathway, including *CD28*, *ZAP70*, *ITK*, *ICOS*, *LAT*, and *PRKCQ* (**Figure 2E**). In particular, we observed exhausted T-cell (Tex) markers, such as TOX, PDCD1, CTLA4, and TIGIT, which were highly expressed in the CD30+ TMF group (**Figure 2E**). Interestingly, TCF7, a marker of proliferation-complete precursors of Tex cells, with genes specifically higher in TCF7+ Tex subsets (*GNG4*, *CD200*, *BTLA*, *IL6R*, and *IGFL2*) *(* 52) have significantly higher expression in CD30+ TMF (**Figure 2E**) (53–55). These findings suggested that CD30+ TMF tumor cells may exhibit a progenitor-exhausted T-cell state. DNMT3A, a key player in *de novo* methylation, exhibited significantly higher expression in CD30+ TMF compared to cALCL (**Figure 2E**), potentially reshaping the DNA methylation atlas to modulate gene expression (56). 

In addition, some disease-specific molecular features are correlated with the clinicopathological findings. We found that neutrophil degranulation was enriched in the cALCL group, consistent with neutrophil infiltration favoring cALCL samples (**Figure 2D**, **Table 1**). CCL17 was more highly expressed in CD30+ TMF compared to the cALCL group (**Figure 2E**). Previous evidence showed CCL17 is associated with pruritus in MF (57), which is consistent with our finding that pruritus favors CD30+ TMF patients (**Table 1**). B cell-associated genes (*MS1A4*, *PAX5*, *CD19*, *CD79A*, *CD79B*, and *CD22*) were differentially expressed in CD30+ TMF compared to cALCL (**Figure 2E**), consistent with the abundance of B cells in CD30+ TMF specimens histologically (**Table 1**). Taken together, the transcriptional profiles of cALCL and CD30+ TMF are unique to each respective disease. The distinct transcriptional programs between cALCL and CD30+ TMF partially explain the differences in their clinicopathologic features.

### 3.3 Distinct regulons drive neoplastic T cell phenotypes in cALCL and CD30+ TMF

**Figure 3:**
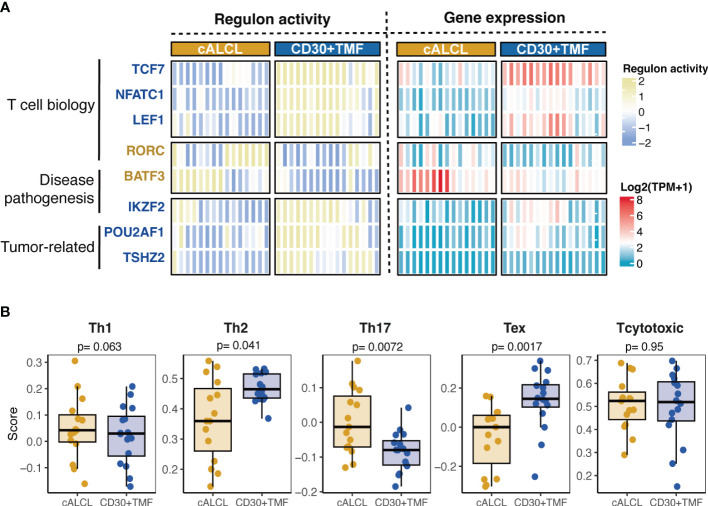
Distinct regulons associated with T cell states. **(A)** Heatmap of regulon-activity scores and expression levels of the transcriptional regulators significantly associated with cALCL or CD30+TMF (regulon activity> 1.6 and p-value< 0.001). On the left side of the heatmap, TCF7, NFATC1, LEF1, IKZF2, POU2AF1, and TSHZ2 were highlighted in dark blue to indicate their enriched regulon activity in CD30+ TMF. RORC and BATF3 were highlighted in dark yellow to indicate their higher regulon activity in cALCL. **(B)** Boxplots showing the enrichment score of different T cell phenotype signatures in cALCL and CD30+ TMF samples determined using the ssGSEA algorithm. Th1, T helper 1; Th2, T helper 2; Th17, T helper 17; Tex, exhausted T cell; TPM, transcripts per million.

We observed that many transcription factors (TFs) were differentially expressed between CD30+ TMF and cALCL (**Supplementary Table 2**). This suggested that CD30+ TMF and cALCL may employ different transcriptional regulators to control the expression patterns of their malignant T cells. Therefore, we selected TF-encoding genes that are differentially expressed between the two diseases and are included in the human TFs reported by Lambert, et al (39). We evaluated the regulatory effect of these TFs on their target genes and identified disease-specific regulators with significant regulon activity when comparing cALCL with the CD30+ TMF group (regulon activity ≥1.6 or ≤-1.6, p-value< 0.001, **Figure 3A**, **Supplementary Table 4**). We found that the significantly activated regulons were involved in T cell biology (RORC, TCF7, NFATC1, and LEF1) or disease pathogenesis (IKZF2 for CD30+ TMF and BATF3 for cALCL) or reported cancer biology (POUZAF1 and TSHZ2) (**Figure 3A**). As a downstream molecule of TCR signaling enriched in CD30+ TMFs (**Figure 2D**), NFATC1 has been reported to promote the proliferation of mature T cells (58), and we found its higher expression and transcriptional activity in CD30+ TMFs (**Figure 3A**). Notably, BATF3 and TCF7 exhibited the most pronounced active regulon activity enriched in cALCL and CD30+ TMF, respectively, as indicated by regulon activity of 1.93 and -1.92 (**Supplementary Table 4**). Moreover, among the activated regulators, the expression of BATF3 and TCF7 was abnormally highest in cALCL and CD30+TMF, respectively (**Figure 3A**). BATF3 has been reported to induce MYC activity and thereby promote tumor growth in anaplastic large cell lymphoma (42), consistent with the MYC targets molecular program enriched in cALCL. In addition, BATF3 drives Th17-skewing phenotype in anaplastic large cell lymphoma (ALCL) (19), and TCF7 confers progenitor exhausted T cell state (54). We, therefore, checked the T cell subtype composition in each sample using the ssGSEA algorithm (32). Accordingly, the Th17 phenotype was significantly enriched in cALCL, and the exhausted T cell phenotype was much more abundant in CD30+TMF (**Figure 3B**). Notably, the comparable abundance of the Th2 state was found in the CD30+TMF and cALCL groups with borderline statistics (**Figure 3B**), correlated with the GATA3, as a master TF of Th2, showed its higher expression in CD30+ TMF (**Figure 2C**), but did not have robust disease-specific transactivation (regulon activity = - 0.83, **Supplementary Table 4**). Therefore, characteristic regulon profiles define CD30+TMF and cALCL.

### 3.4 Different tumor microenvironments and clinicohistological correlation in cALCL and CD30+ TMF

The interactions and intricate cross-talk between malignant T cells and the tumor microenvironment (TME) have garnered significant attention recently. Next, we sought to characterize the detailed cell subpopulations in the TME of cALCL and CD30+ TMF samples. As both diseases are lymphomas derived from mature T cells, the assessed T-cell abundance primarily refers to malignant T-cells rather than reactive T-cells. Therefore, we excluded T-cell subpopulations and included other immune cells and stromal cells involved in skin lesions from 64 cell types in the xCell software for TME assessment.

**Figure 4:**
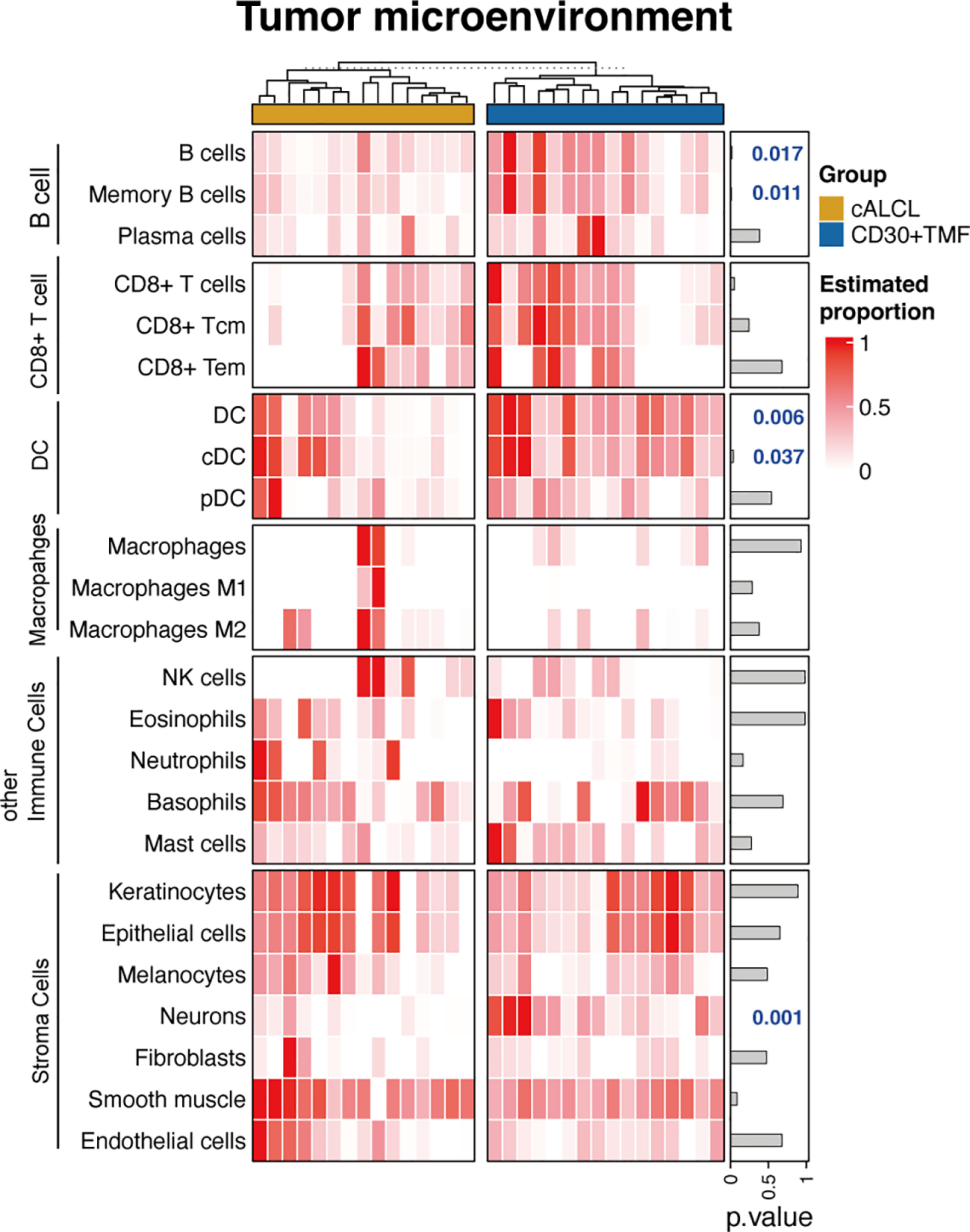
Tumor microenvironment correlated with clinicopathological information. Heatmap of the estimated proportions of different cell subtypes determined by the xCell software. Tcm, central memory T cell; Tem, effector memory T cell; DC, dendritic cell; cDC, conventional dendritic cell; pDC, plasmacytic dendritic cell; NK, natural killer.

Our results demonstrated that CD30+ TMF has an enrichment of B cells, specifically memory B cells, compared to cALCL (B cells: p-value = 0.017; memory B cells: p-value = 0.011) (**Figure 4**), consistent with more CD30+ TMF specimens showed CD20-positive immunostaining compared to cALCL as an immunophenotype (**Table 1**). Correspondingly, the expression of B cell markers and the B cell activation process were found to be enriched in CD30+ TMF groups (**Figures 2D, E**). Furthermore, dendritic cells (DCs) were found to be more abundant in CD30+TMF compared to cALCL (**Figure 4**). This may help explain the high level of epidermotropism observed in CD30+TMF, as the core cell type in Pautrier’s microabscesses is Langerhans cells, which are immature dendritic cells (59). We observed the presence of neurons in CD30+ TMF samples that were absent in cALCL lesions (**Figure 4**). This finding suggests a possible association with the pruritus experienced by most patients in the CD30+TMF group (**Table 1**). Taken together, we observed distinct tumor microenvironment features in CD30+ TMF that correlated with its histopathologic findings, but the TME features of cALCL appear to be non-specific with neutrophil abundance shown in a proportion of cALCL (**Figure 4**).

### 3.5 BATF3 and TCF7 immunostaining pattern for distinguishing cALCL from CD30+ TMF lesions

**Figure 5:**
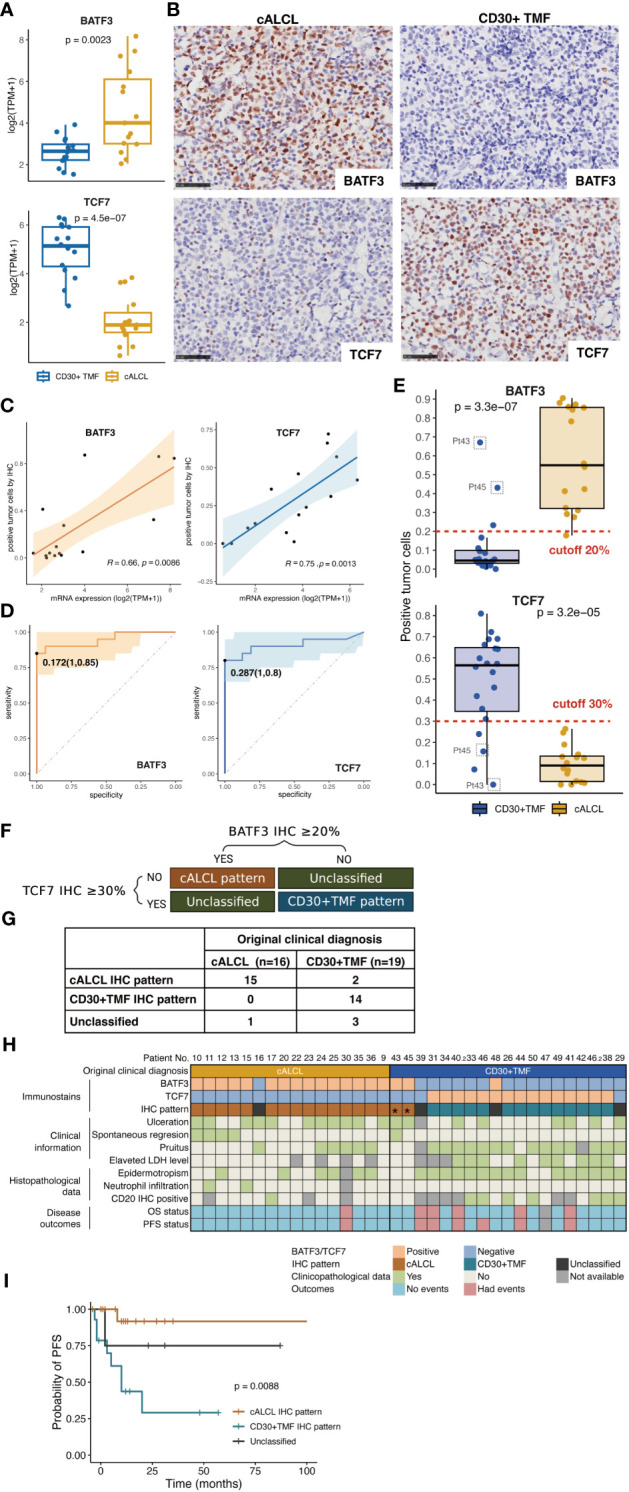
Generation of the IHC algorithm. **(A)** Comparison of expression levels of BATF3 and TCF7 in cALCL and CD30+TMF group, by Wilcoxon test. **(B)** Representative images of immunohistochemical staining of BATF3 and TCF7 in biopsy specimens from CD30+ TMF and cALCL patients. Original magnification ×400. Scale bar = 50 μm. **(C)** Correlation between mRNA and IHC expression of BATF3 and TCF7. Statistical significance was determined by Spearman’s correlation coefficient for BATF3 and Pearson correlation coefficient for TCF7. **(D)** ROC curve analysis for IHC expression showed that the most appropriate threshold for BATF3 is 0.172 with sensitivity (1.0) and specificity (0.85). The most appropriate threshold for TCF7 is 0.287 with maximized sensitivity (1.0) and specificity (0.8). **(E)** Distribution of immunostaining positivity in the CD30+ TMF and cALCL groups. Top panels, the cALCL group showed higher positivity for BATF3 immunostaining compared to the CD30+TMF group, with 20% being the best cutoff to separate the two diseases. Bottom panels, the CD30+TMF group showed higher positivity for TCF7 immunostaining compared to the cALCL group, with 30% being the best cutoff to separate the two diseases. **(F)** IHC patterns were generated by BATF3 and TCF7 immunostains. **(G)** Comparison of the IHC pattern and the original clinical diagnosis. **(H)** Heatmap representation of BATF3 and TCF7 immunostaining, IHC pattern, and comparative clinicopathological information. **(I)** PFS curves were classified by IHC patterns. PFS, progression-free survival.

Next, we aimed to identify differentially expressed biomarkers between cALCL and CD30+ TMF based on their distinct gene expression profiles, and to identify these markers through cost-effective IHC staining to facilitate the diagnostic process. IHC candidates were selected based on sufficient expression levels and significant correlation (Wilcoxon text, p<0.01) with the respective disease. Commercial antibodies against the selected molecules should be available and show reactivity on FFPE tissue. We finally identified BATF3 and TCF7 as potential markers for cALCL and CD30+ TMF, respectively. The transcript levels of the two markers showed robust differences between the two tumor lesions (**Figure 5A**). Their IHC staining was specific and clear for lymphoid cells with minimal background (**Figure 5B**). The positive rate of immunostaining was significantly correlated with the corresponding mRNA expression levels for both BATF3 (R= 0.66, p= 0.0086) and TCF7 (R= 0.75, p= 0.0013) (**Figure 5C**). We determined the cutoffs for positivity based on the percentage of staining that effectively separated the diseases with minimal error rates, as estimated by the area under the receiver operating characteristic (ROC) curve. The optimal cutoffs were 17.2% positivity for BATF3 (with 1.0 specificity and 0.85 sensitivity) and 28.7% positivity for TCF7 (with 1.0 specificity and 0.8 sensitivity) (**Figure 5D**). For practical clinical use, we approximated the cutoffs to 20% for BATF3 and 30% for TCF7 (**Figure 5E**).

Based on these cutoffs, we established an IHC algorithm for differentiating cALCL and CD30+ TMF: 1) positive BATF3 expression (BATF3 IHC ≥ 20%) and negative TCF7 expression (TCF7 IHC < 30%) indicated the “cALCL pattern”; 2) positive TCF7 expression (TCF7 IHC ≥ 30%) and negative BATF3 expression (BATF3 IHC < 20%) defined the “CD30+ TMF pattern”; 3) all other combinations were categorized as “unclassified” (**Figure 5F**). Among the 36 samples, 15 cALCL samples and 2 CD30+ TMF samples showed the “cALCL pattern”, 15 CD30+ TMF samples showed the “CD30+ TMF pattern”, and 4 were classified as “unclassified” (**Figure 5G**). **Figure 5H** shows the original clinical diagnoses and IHC patterns for all samples, along with significant clinicopathologic features that may aid in diagnosis. We found that the IHC patterns were associated with the disease PFS (**Figure 5I**, p= 0.0088).

Interestingly, two samples (Pt43 and Pt45) that were initially diagnosed as CD30+ TMF exhibited the “cALCL pattern” in our analysis (**Figure 5H**, indicated by asterisks). These samples showed significantly high BATF3 expression and low TCF7 expression (**Figure 5E**, indicated by gray squares). For further investigation, we carefully reviewed the clinical histology of these cases. Pt43 and Pt45 presented with patches and plaques distributed over their bodies approximately 10 and 5 years ago, respectively. Subsequently, nodules with ulcers appeared 3 and 6 months before their clinic visits, respectively. Skin biopsies were performed on the nodular lesions, followed by a follow-up period of 18 and 40 months, during which no disease progression was observed and a partial response to treatment was achieved (**Figure 5H**). Clinicopathologically, these cases showed ulceration, normal LDH levels, and the absence of pruritus and epidermotropism, CD20-negative immunostaining, suggesting that these cases may represent cALCL lesions (**Figure 5H**). Considering the clinicopathologic data and the IHC pattern, we concluded that the diagnosis of both patients should be revised to cALCL arising in the context of MF. Therefore, BATF3 and TCF7 can serve as reliable immunohistochemical markers to differentiate these two entities.

## 4 Discussion

**Figure 6:**
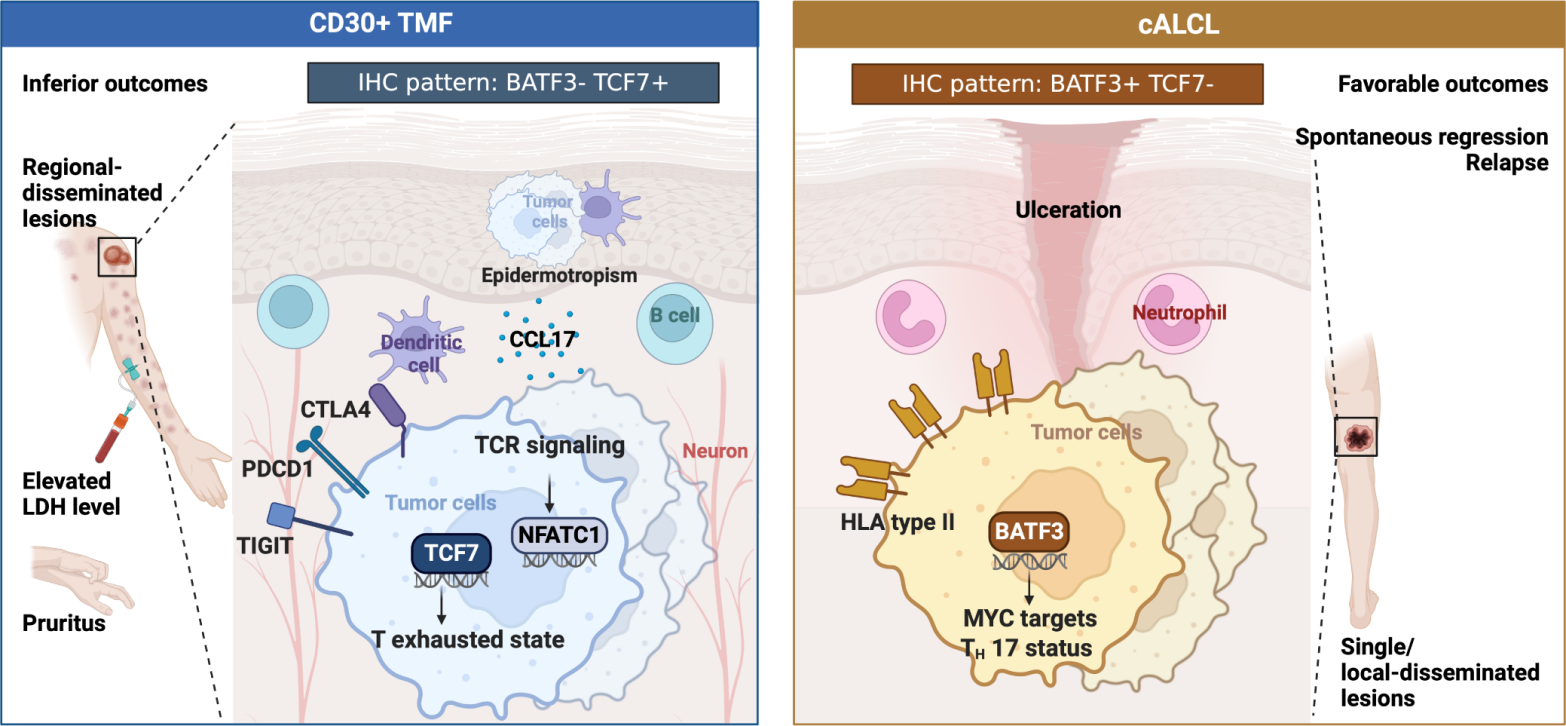
Schematic summary.

Distinguishing between cALCL and CD30+ TMF is challenging, but critical, as they show distinct progression and require different patient management. Applicable molecular diagnostic tools are lacking, in part due to the absence of whole transcriptome comparison and screening. In the present study, we first compared the transcriptional atlas between cALCL and CD30+ TMF, revealing distinct biological programs associated with their clinicopathological features (**Figure 6**). Based on the specific expression profiles, we generated innovative IHC patterns using two panels of antibodies to discriminate cALCL from CD30+ TMF lesions.

Clinically, CD30+ TMF patients were observed to have regionally disseminated lesions with disease progression and poor outcomes. Elevated LDH levels and pruritus were common in these patients, with some experiencing severe pruritus that disrupted their sleep. Pruritus significantly affects the health-related quality of life (HRQoL) in CTCL patients (60, 61) and should be a critical consideration in clinical management. Histopathologically, CD30+ TMF cells were epidermotropic with greater infiltration of dendritic cells and B cells. Molecularly, CD30+ TMF showed a progenitor-exhausted T-cell status driven by TCF7 and enriched TCR signaling pathways with NFATC1 activation. Neuron infiltration and higher CCL17 expression may contribute to the pruritus symptom (**Figure 6**).

In contrast, cALCL patients showed favorable outcomes with lesions found as solitary or locally disseminated occurrences. Spontaneous regression and relapse were more frequent in cALCL. Histologically, a proportion of cALCL biopsies showed an abundance of neutrophils, a feature not observed in CD30+ TMF lesions. cALCL cells exhibited natural BATF3 upregulation with higher regulon activity compared to CD30+ TMF, contributing to the enrichment of MYC targets and the Th17 phenotype. In addition, cALCL cells expressed higher levels of HLA type II genes than normal CD4+ T cells, which differed from the CD30+ TMF groups (**Figure 6**).

Previous studies support our findings regarding a subset of MF exhibiting exhausted T-cell profiles (33, 62). We identified a TCF7+ Tex signature enriched in TMF, which is consistent with previous reports of TCF7+ malignant T cells in the aggressive molecular subtype of CTCL that included all TMF cases in their cohort (63). Previous studies have reported an increase in B-cell abundance in MF during disease progression, which is consistent with our findings of B-cell infiltration in the CD30+ TMF group (40, 64, 65).

On the other hand, we identified aberrant expression of BATF3 in cALCL with the enrichment of Th17 state and MYC targets, which is supported by the reports that BATF3 aberrantly expressed to drive ALCL survival by inducing MYC activation and Th17 phenotype (19, 42). Notably, HLA class II genes were highly expressed in cALCL, compared to normal CD4+ T cells and CD30+ TMF lesions. HLA class II expression has also been observed in various tumors, including hematologic malignancies, and has been associated with improved outcomes or response to checkpoint blockade (50, 51, 66). The precise mechanism remains unknown, but it is hypothesized that the interaction between HLA type II-expressing lymphoma cells and CD4+ T cells may contribute to tumor immunity in cALCL, potentially contributing to spontaneous tumor regression and improved outcomes. In contrast, IKZF2 transactivation was enriched in CD30+ TMF, and IKZF2 has been reported to downregulate HLA type II genes in MF cells, which may help to CD30+ TMF cells escape the anti-tumor immunity (48). Further research is warranted to explore the expression and function of HLA type II genes in both diseases.

The comparative transcriptional analysis also identified a set of differentially expressed genes that show promise as candidate markers for distinguishing cALCL from CD30+ TMF. We developed a novel diagnostic model incorporating BATF3 and TCF7 immunostains that holds substantial clinical value. Reclassification of samples using the IHC model provided a more significant prognostic value of disease diagnosis compared to the initial clinical diagnosis. Only 8.3% of the samples remained “unclassified”, while the majority could be accurately classified as either “cALCL pattern” or “CD30+ TMF pattern”, providing valuable guidance for clinical diagnosis. The use of the 2-gene IHC model enabled the successful identification of cALCL lesions occurring in the context of MF as a “cALCL pattern”, which successfully reclassified two patients initially diagnosed with TMF as having cALCL with MF. We believe that this IHC model holds promise as a potential diagnostic tool, particularly when dealing with identical lesions that develop in the setting of pre-existing MF disease or when the clinical history is equivocal. However, due to the rarity of both cALCL and CD30+ TMF disease, our IHC algorithm was generated based on 36 samples. Therefore, further research with larger cohorts from multiple centers is essential to improve the accuracy and enhance the clinical utility of this IHC algorithm.

Epigenetic modulators are increasingly recognized as essential factors that shape gene expression and offer opportunities for intervention. Multiple lines of evidence emphasize the crucial role of epigenetics in the development of CTCL. Unfortunately, there are lack of sufficient epigenome data from cALCL and CD30+ TMF for direct analysis. T cell-associated transcription factors such as LEF1, TCF7, and GATA3 were decreased in cALCL in comparison to CD30+TMF in our dataset, which are possibly altered by the suppressive trimethylation of histone H3 lysine 27 in cALCL (67, 68). These findings emphasize the necessity of a wide-ranging multi-omics investigation, including epigenetic profiles, accurate transcriptomic atlases, and a genomic landscape.

Of note, our bulk RNA-seq data indicated an enrichment of extracellular matrix (ECM)-associated gene sets in cALCL lesions compared to CD30+ TMF (**Supplementary Table 3**), while a recent study by Choi, et al. using spatially resolved transcriptomics has identified that the signature of ECM remodeling and fibroblasts are enriched in CD30+ regions within TMF lesions when compared with those in cALCL lesions (69, 70). Our study examined the cellular proportions in whole skin tumor specimens, and showed no difference in fibroblast abundance between the two entities (**Figure 4**), whereas Choi, et al. focused on tumor cells and their surrounding cells, and they reported intratumoral heterogeneity between CD30- and CD30+ regions in both entities (69). This discrepancy in the selection of area-of-interest may cause the difference of the findings in the cellular components of ECM between the two studies. Likewise, genes comtributing to tissue fibrosis, including TGF-β and AHR, which were highly expressed in CD30+ regions within TMF compared to those in cALCL in Choi, et al., did not show differential expression between cALCL and CD30+TMF in our dataset (**Supplementary Table 2**). Considering the complexity of tumor microenvironment shaping, more comprehensive investigations with advanced techniques and high resolution are required in larger cohorts.

In conclusion, our study has shed light on the distinct molecular programs associated with the different clinicopathologic features of CD30+ TMF and cALCL. CD30+ TMF shows a TCF7+ exhausted T-cell state with B-cell infiltration, whereas cALCL shows aberrant BATF3 expression with HLA type II expression. The 2-gene IHC patterns we developed are readily applicable in the clinical setting and may help to differentiate between these two entities, particularly in cases with large CD30+ cell-infiltrated lesions associated with MF disease. These findings significantly advance our understanding of the molecular differences between the two diseases and pave the way for improving diagnostic accuracy in clinical practice.

## Data availability statement

The datasets presented in this study can be found in online repositories. The names of the repository/repositories and accession number(s) can be found in the article/**Supplementary Material**.

## Ethics statement

The studies involving humans were approved by Peking University First Hospital. The studies were conducted in accordance with the local legislation and institutional requirements. The participants provided their written informed consent to participate in this study.

## Author contributions

PL: Investigation, Data curation, Methodology, Visualization, Writing – original draft. FL: Data curation, Writing – review & editing. XL: Data curation, Writing – review & editing. JS: Data curation, Writing – review & editing. YW: Writing – review & editing, Project administration, Supervision.
